# Personality change through a digital-coaching intervention: Using measurement invariance testing to distinguish between trait domain, facet, and nuance change

**DOI:** 10.1177/08902070221145088

**Published:** 2022-12-14

**Authors:** Gabriel Olaru, Mirjam Stieger, Dominik Rüegger, Tobias Kowatsch, Christoph Flückiger, Brent W Roberts, Mathias Allemand

**Affiliations:** 1 7899 Tilburg University, Tilburg, Netherlands; 2 30744 Lucerne University of Applied Sciences and Arts, Luzern, Switzerland; 3 ETH Zurich, Zurich, Switzerland; 4 University of St. Gallen, St.Gallen, Switzerland; 5 27217 University of Zurich, Zurich, Switzerland; 6 University of Illinois, Urbana-Champaign, IL, USA; 7 University of Tübingen, Tübingen, Germany

**Keywords:** personality change, facets, measurement invariance, digital-coaching intervention, personality coach

## Abstract

Recent intervention research has shown that personality traits can be modified through psychological interventions. However, it is unclear whether reported effects represent changes in the trait domain or only some facets or items. Using data (*N* = 552) from a recent intervention trial, the present study examined the effects of a digital-coaching intervention on self- and observer-reported personality facets and items. We focused on participants who wanted to decrease in Negative Emotionality, increase in Conscientiousness or increase in Extraversion. We used measurement invariance testing to examine which level of the trait domain hierarchy changed during the intervention. For the self-reports, we found some heterogeneity in the effects on all three trait domains, but most notably Extraversion and Conscientiousness. Specifically, participants reported to increase strongly on sociability (Extraversion), and moderately on productiveness and organization (Conscientiousness), but not on the other facets of these trait domains. Observers generally reported small but non-significant changes, with no scalar invariance violations except for Extraversion. Overall, this suggests considerable heterogeneity in intervention-related personality change that can be overlooked if only focusing on the trait domain level. We discuss the relevance of measurement invariance testing and measurement approaches for personality development and intervention research.

## Introduction

Recent intervention work has shown that personality traits are more plastic than previously thought. Already having the goal to change one’s personality can set in motion change processes that can lead to personality change (Hudson et al., 2020). Furthermore, being motivated to change and actively working on personality change through regular engagement in small behavioral activities and other micro-interventions can bring about personality change (Hudson et al., 2019; Stieger, Wepfer, et al., 2020). One way to deliver personality change interventions to people is through smartphones applications. Digital-coaching apps are specifically useful to motivate, support, and accompany people in their daily change efforts in everyday contexts (Allemand & Flückiger, 2022). A recent randomized controlled trial, which provides the data for the present study, tested the effectiveness of three-months of personality change coaching with a digital application in a large sample (Stieger et al., 2021). The findings from that trial provide the strongest evidence to date that broad personality traits can be changed through intervention in a nonclinical sample. However, it is unclear how the narrower facets and nuances (i.e., narrow subtraits represented by individual items; McCrae & Mõttus, 2019) change and whether they change in the same way as the overarching trait domains. Building on recent advances in personality conceptualizations and research demonstrating the incremental value of facets and nuances for studying personality development (Mõttus & Rozgonjuk, 2021; Olaru et al., 2019; Soto et al., 2011) and the prediction of important outcomes (Danner et al., 2021; Seeboth & Mõttus, 2018; Stewart et al., 2021), we examined the effects of the digital-coaching intervention on personality facets and nuances in both self- and observer-reports.

### Personality change interventions

The most common evidence that personality can be changed comes from studies on clinical interventions (e.g., Bagby et al., 1995; De Fruyt et al., 2006; Roberts et al., 2017; Santor et al., 1997; Sauer-Zavala et al., 2020; Stieger et al., 2022; Tang et al., 2009). These studies often incorporated personality trait measures in addition to clinical measures to assess pre- and post-treatment effects of clinical, psychopharmacological, and psychotherapeutic interventions (see Jackson et al., 2021 for a review). Based on this literature, there is evidence that clinical interventions and psychotherapy with the goal to target mental health problems and individual functioning also influence personality traits as “side effects.” For example, a recent study tested the effects of a 4-week intervention on clinical states (e.g., stress or depression) and personality traits in a sample of substance use patients across 28 weeks (Stieger et al., 2022). The results indicated large changes of the clinical states and the traits that occurred rapidly during the intervention. A large-scale meta-analysis of 207 clinical intervention studies found broad evidence for marked changes in personality traits over an average time interval of 24 weeks (Roberts et al., 2017). Neuroticism was the primary trait showing decreases as a result of therapy, followed by increases in Extraversion. Most changes in personality traits occurred in the first couple of weeks of therapy and plateaued after 8–10 weeks. Such a pattern of relatively rapid change contrasts the slow developmental change processes typically observed in longitudinal observational studies (Roberts et al., 2006). The review also found that the trait changes persisted in longitudinal follow-ups beyond the course of intervention. Moreover, in psychotherapy, there is a lasting research tradition to describe conditions where patients are able to explore and change their personality independent of particular therapeutic techniques most likely reflecting shared interventional principles across different orientations (e.g., Kramer et al., 2020; Norcross & Goldfried, 2019; Roberts et al., 2017; Rogers & Dymonds, 1954).

Further evidence for personality change through interventions comes from recent studies in non-clinical populations (e.g., Allemand et al., 2022; Hudson & Fraley, 2015; Hudson et al., 2019; Stieger, Wepfer, et al., 2020; Stieger et al., 2021; see Jackson et al., 2021 for a review). For instance, a 16-week intensive longitudinal study demonstrated that regularly generating specific implementation intentions (i.e., “if-then” plans) for personality change goals was associated with trait changes (Hudson & Fraley, 2015). In addition, a 15-week intensive longitudinal study found that active and successful implementation of change behaviors was a successful strategy for changing personality traits (Hudson et al., 2019). Other studies used coaching approaches to elicit personality change. For example, in a 10-week coaching program, participants worked with a coach to identify roadblocks of behaving in a trait-consistent manner, as well as developing a more elaborate strategy that targeted specific traits (Allan et al., 2018; Martin et al., 2014). Participation in the coaching program was associated with positive personality change. A recent study explored the effects of two-bottom up intervention approaches (Massey-Abernathy & Robinson, 2021). The first approach included a 5-week behavioral activation training and demonstrated increases in facets of Conscientiousness in response to the training. The second approach included a 12-week coaching intervention based on instructional feedback and monitoring to target personality traits. This coaching intervention led to changes in four of the Big Five traits. Other studies used digital personality change interventions. A two-week intervention study tested the effectiveness of a digital-coaching intervention using text messaging services to either target Self-discipline, a facet of Conscientiousness, or Openness to Action, a facet of Openness to Experience (Stieger, Wepfer, et al., 2020). People who chose to become more self-disciplined showed a greater increase in Self-discipline than Openness to Action, the reverse was true for those who wanted to increase in Openness to Action. The observed changes were maintained at the follow-up 2- and 6- weeks later.

A recent randomized controlled trial drew on the experience of this first digital intervention and extended it with the use of a smartphone application to deliver psychological micro-interventions. The effectiveness of the 3-month digital-coaching intervention on personality trait changes was examined in a large non-clinical sample (Stieger et al., 2021). The results showed that those individuals who received the digital-coaching reported greater changes than a wait-list control group. In addition to the results based on self-reports, observers such as friends, family members, or intimate partners also detected significant, albeit smaller, changes for those desiring to increase on a trait but not for those desiring to decrease on a trait. Finally, the findings indicated that the personality trait changes in response to the digital-coaching intervention persisted in a three-month follow-up.

In summary, existing clinical and non-clinical intervention research provides consistent evidence that personality traits can be changed in desired directions through psychological treatments. However, previous studies on personality change through interventions focused solely on broad trait domain level (but see Stieger, Wepfer, et al., 2020). Trait domains are very heterogeneous constructs encompassing many narrower traits (e.g., facets, nuances), which might respond differently to the intervention efforts. As these narrower traits are also not equally predictive of relevant life outcomes (e.g., Danner et al., 2021; Stewart et al., 2021) and are not equally desirable to change for individuals (Sun & Goodwin, 2020), understanding which components of the trait domain have changed due to the intervention is also relevant to judge the indirect effects of interventions on life outcomes though personality traits.

### Intervention effects on personality facets and nuances

Studies on personality development have found considerable heterogeneity in the age differences of the Big Five facets (Chopik, 2016; Olaru et al., 2018; Soto et al., 2011) and nuances (Hang et al., 2021; Mõttus & Rozgonjuk, 2021). Some of the mixed findings on developmental trends of Extraversion and Openness can be explained by some components increasing whereas others stay stable or decrease in their mean-levels (e.g., Olaru et al., 2018). Focusing solely on the trait domain level will thus only provide an aggregate of potentially diverging change patterns, and therefore under- or overestimate change in narrower traits. Although personality intervention efforts are generally tailored to affect the entire trait domain (e.g., Stieger et al., 2018, 2021) or a specific facet (e.g., self-discipline; Stieger, Wepfer, et al., 2020), some facets or nuances of the trait domain may change more during intervention efforts than others. For example, many personality intervention studies use—among others—behavioral activation or implementations intentions (i.e., “if-then plans”) to help participants form habits that should affect the underlying traits in a bottom-up fashion (Allemand & Flückiger, 2017). Using these approaches, participants may find it easier to increase their sociability, but not necessarily their cheerfulness—both of which are facets of Extraversion. Another reason for heterogeneous change patterns may be that participants in the intervention focused on some facets of the trait domain more than others, for example, working more on their assertiveness than their gregariousness. Furthermore, the self-reflection activities (or generally participating in trait-related activities) may help participants gain more insight into themselves and thus affect the way they interpret and respond to the self-report measures. Examining the effect of interventions at a more fine-grained level is important because one argument made in favor of personality interventions is the association of personality traits with relevant life outcomes (e.g., Bleidorn et al., 2019; Soto, 2019). Because the associations between life outcomes and facets (e.g., Danner et al., 2021; Stewart et al., 2021) or nuances (e.g., Seeboth & Mõttus, 2018; Stewart et al., 2021) can differ from the effects found at the trait domain level, it is relevant to know whether the entire trait domain changed or only some components thereof.

### Measurement invariance testing as a tool to examine homogeneity in trait changes

One of the most common approaches used to examine if changes or differences are only observable at the item, facet or trait domain level—or any latent construct in general—is measurement invariance testing (e.g., Chen, 2007; Little, 2013; Meredith, 1993). In general, this is done by comparing a model in which all item intercepts are allowed to vary across time or (age) groups to a model in which only the latent trait mean is allowed to vary. A substantial increase in model misfit would suggest that the latent trait alone cannot account for all changes or differences in the indicators across time or (age) groups. This approach has been frequently used in personality development research before factor means were compared across time or age (e.g., Allemand et al., 2007; Nye et al., 2016). However, this approach is also relevant for personality change in the context of intervention studies (for a discussion see, Könen & Karbach, 2021). In the following, we provide a more in-depth explanation of this approach.

Measurement invariance is generally tested by comparing the model fit of models with increasingly strict parameter constraints, starting with no equality constraints across time and/or groups (i.e., configural invariance), constraining the factor loadings to equality across time and/or groups (i.e., metric invariance), and subsequently additionally constraining indicator intercepts to equality across time and/or groups (i.e., scalar invariance). In the configural and metric invariance model, factor means are usually constrained to zero by default (but see Little et al., 2006 for alternative approaches) and indicator intercepts are allowed to vary. When applying scalar measurement invariance constraints (i.e., equal indicator intercepts), the factor means are freed and estimated. If the indicator-level differences across time and/or groups are proportional to the factor loadings (e.g., items or facets that are more central change/differ most strongly; or they change to the similar degrees if factor loadings are somewhat equivalent), they can be summarized by the differences in the factor mean and scalar measurement invariance should hold. Conversely, if the differences or changes at the item or facet level cannot be explained by differences or changes in the overarching factor, scalar measurement invariance should be violated. For example, Estrada and colleagues (2015) used measurement invariance testing to examine if changes in a raven progressive matrices, abstract reasoning, verbal reasoning, and spatial reasoning test during a cognitive training could be interpreted as changes in a common general intelligence factor. The lack of scalar measurement invariance across time suggested that the training resulted in differential changes in the specific abilities that could not be accounted for by the general factor. Although the lack of scalar measurement invariance does not indicate a lack of gains due to the training, it does affect the way in which these gains should be interpreted: As improvements in specific abilities, but not necessarily (or only partly) in general intelligence or the overarching trait domain.

In the context of higher-order personality traits, measurement invariance testing can thus be used to identify whether mean-level change or differences are unique to specific items or facets, or similar across the entire trait domain (for an example with higher-order personality trait models across age, see Olaru et al., 2018). We suggest measurement invariance testing as an approach to investigate these patterns, as it provides a straightforward way to test at which level of the trait hierarchy potential changes are located.

### The present study

The goal of the present study was to expand upon the results from the randomized controlled trial (Stieger et al., 2021) with a more fine-grained picture on the effects of the digital-coaching intervention on personality facets and nuances—in both self- and observer-reports. We used measurement invariance testing to examine whether the broad interventions approach affected all components of the trait domain similarly, only some facets, or only some nuances. Participants were able to choose the direction of personality trait change. To achieve sufficient power and sample size for the models, we focused on the five largest change goal groups: Decrease in Negative Emotionality, increase in Conscientiousness, or increase in Extraversion. We used participants working on a different trait domain as an active control group for the change goal groups, to ensure that change was unique to the target intervention groups.

## Method

This research was conducted according to the Declaration of Helsinki and the full study protocol was approved by the Ethics Committee of the Philosophical Faculty of the University of Zurich (No. 17.8.4; Date of approval: August 31st, 2017). The analyses were exploratory and not pre-registered. All analyses were run in R version 4.0.4 (R Core Team, 2021) with the R packages *faux* (DeBruine, 2021), *haven* (Wickham et al., 2022), *lavaan* (Rosseel, 2012) and *psych* (Rosseel, 2020).

### Participants and procedure

Data came from a digital personality change intervention trial (Stieger et al., 2021). A detailed report of the study design, sample size calculation, recruitment process, and measures can be found in the intervention study protocol (Stieger et al., 2018). Personality was measured at three time points, at the beginning of the intervention (i.e., pre-test), at the end of the intervention 3 months later (i.e., post-test), and again 3 months after the intervention ended (i.e., follow-up). Of the 1523 participants who filled out the pre-test, we only included the 552 participants (*n* = 321 female; age: *M* = 25.35 years; *SD* = 7.67)^1^ who also filled out the post-test. Participants were able to choose their desired Big Five trait domain and direction of change for the intervention (with the exception of an increase in Negative Emotionality). Of the nine resulting change goal groups, we focused on the three largest groups in the current study to achieve sufficient sample size for the model estimation. These three change goal groups included participants who desired to decrease in Negative Emotionality (*n* = 168; 30.4%), increase in Conscientiousness (*n* = 140; 25.4%), or increase in Extraversion (*n* = 110; 19.9%). The next largest change goal group—which we did not examine in this study—desired to increase in Openness (*n* = 44; 8.0%)^2^. For each of these groups, we used all other participants that chose a different Big Five factor in their change goal as active control group.

Personality self- and observer-reports were administered at three time points, at the beginning of the intervention (i.e., pre-test), at the end of the intervention 3 months later (i.e., post-test), and again three months after the intervention ended (i.e., follow-up). Participants were asked to contact at least three close friends, family members, and/or romantic partners at the beginning of the study to acquire observer-reports. We only used observer-reports if they were provided by the same observer in at least two measurement occasions. Observer-reports were available for a total of 342 targets. Observers reported how well they knew the target on a scale from 1 = very distant to 7 = very close with an average score of 6.24 (SD = 0.91). The number of participants with self- and at least one observer-report per change goal group and measurement occasions is presented in Table 1 (respondents were required to answer all items of the questionnaire if they submitted at least one response).

**Table 1.** Available cases per change goal group, measurement occasion, and data source.

Change goal group	Pre- & post-test		Follow-up	
	Self	Observer	Self	Observer
Negative emotionality (−)	168	131(1.64)	136	105(1.60)
Conscientiousness (+)	140	131(1.53)	101	86(1.33)
Extraversion (+)	110	76(1.38)	96	54(1.42)

*Note*. (−) = decrease; (+) = increase; For the observer reports, numbers in parentheses indicate the average number of observers per participant.

### Personality change intervention

The digital-coaching intervention was conducted using the smartphone application PEACH (Android and iOS), a digital coach that automatically guides and supports people in achieving their personality change goals (Stieger et al., 2018). People interacted daily with a chatbot and received information, education, feedback, encouragement, and support. Conceptually, the digital-coaching intervention was developed based on a common change factors model (Allemand & Flückiger, 2017, 2022). The idea is that four common change factors provide useful heuristic principles for personality change interventions. First, intervention efforts should actuate discrepancy awareness, which refers to the idea that when people should be made aware of how they want to be different than they are. Second, interventions should activate people’s capabilities, preferences, and resources to initiate and sustain positive-feedback loops and expectations. Third, interventions should target reflective processes, with the goal of helping people better understand their underlying assumptions, expectations, and motivations to promote insight. Fourth, interventions should target behavioral processes of mastery in action, with the goal of helping people explore and practice new behaviors and gradually increase their engagement in new activities and behaviors outside their “comfort zone.” The main goal of the change factor perspective is to optimally integrate all four change factors to maximize intervention effects (Allemand & Flückiger, 2017, 2022). Details about the intervention features, contents, and techniques and the PEACH application are reported elsewhere (Stieger et al., 2018, 2021).

### Measures

#### Self-reported personality traits

Personality trait domains, facets, and nuances were measured with the Big Five Inventory-2 (BFI-2; Soto & John, 2017a). The BFI-2 is a 60-item measure of the Big Five personality trait domains and 15 corresponding facets (i.e., three facets per trait domain). Each facet is measured by four items, two of which are negatively keyed. Participants responded to the items on a 5-point Likert scale ranging from *1 = strongly disagree* to *5 = strongly agree*.

#### Observer-reported personality traits

Observer ratings on the personality traits were assessed with the observer-report version of 30-item BFI-2-S (short version of the BFI-2; Soto & John, 2017b). The BFI-2-S measures the same 15 BFI-2 facets, but with only two items per facet. For each facet, one item is negatively keyed. Observers responded to the items on a 5-point Likert scale ranging from *1 = strongly disagree* to *5 = strongly agree*. For each participant (i.e., target) we combined all available reports from different observers by computing the average of responses for each item.

### Statistical analysis

#### Measurement models

We modeled each facet and trait domain as multi-group longitudinal correlated factor models with three measurement occasions (i.e., pre-test, post-test, and follow-up) and two groups (i.e., target change goal vs. control group). We estimated the models for each facets and each trait domain separately to ensure that the other items did not affect the measurement invariance tests. For the facet models, factors loaded on the corresponding four (self-report) or two (observer-report) items. For the trait domain models, we used three facet parcels as indicators (i.e., the average scores across the items of each facet) for the trait domain models, as higher-order models led to convergence issues with the small samples available for some change groups. To scale the factors, we constrained the first factor loading to 1 and factor means to 0. Residuals of the same item were allowed to correlate across time (Little, 2013).

All models were estimated with the lavaan package in R (Rosseel, 2012). We used *full maximum likelihood estimation* to address missing values in the data. We evaluated overall model fit with a combination of the comparative fit index (CFI), the root mean square error of approximation (RMSEA), and the standardized root mean square residual (SRMR) based on common standards (acceptable/good fit: CFI ≥.90/.95; RMSEA ≤.08/.06; SRMR ≤.08/.06; Bentler, 1990; Hu & Bentler, 1999).

#### Measurement invariance

**Figure 1.**
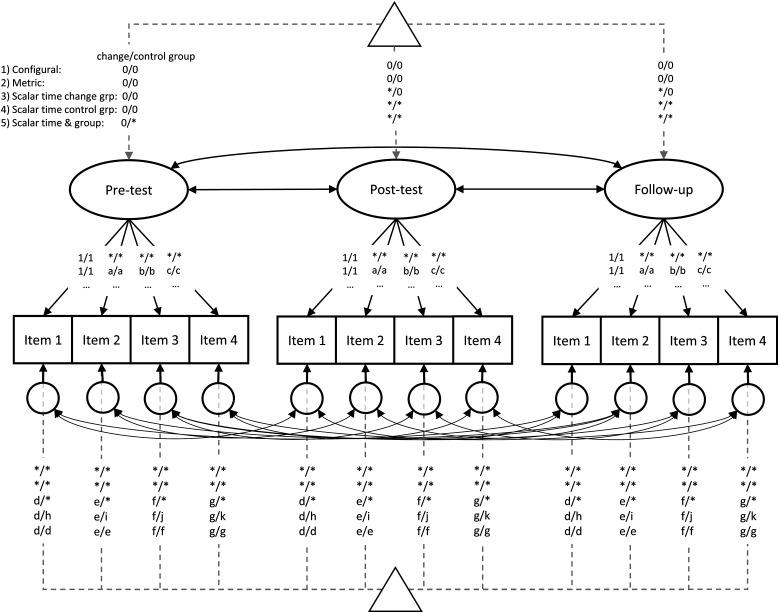
Longitudinal correlated factor model with measurement invariance constraints across time and groups. *Note*. Squares represent observed variables, circles represent latent variables, triangles represent mean, and intercept parameters. Solid single headed arrows indicate loadings, double-headed arrows correlations. Labels on the arrows represent parameter constraints for (1) configural, (2) metric and (3–5) scalar measurement invariance. Labels before the slash (/) show the change goal group constraints, labels after the slash show constraints for the control group. 0 or 1 = constrained to 0 or 1; a to g = constrained to equality across time and groups; * = estimated freely. Depicted is a model for the self-reported facets with four items. For the trait domain models, three facet parcels were used as indicators (but the same constraints apply).

We tested for measurement invariance across time and groups by comparing model fit between increasingly constrained models (Little, 2013; Meredith, 1993). We based our measurement invariance levels on previous studies that discussed or used measurement invariance testing in intervention studies (Estrada et al., 2015; Könen & Karbach, 2021), but made some modifications. Namely, we first tested for the comparability of factor means across time within each group, instead of across groups at each time point. We did so because we were primarily interested in whether mean-level changes within the change goal groups could be summarized by the overarching factor. Adding constraints across groups first would have prevented us from identifying whether measurement invariance violations across time were due to the change goal or comparison group. We compared (1) a model with no additional parameter constraints (i.e., configural invariance) across time and groups, (2) a model with equality constraints on factor loadings (i.e., metric invariance) across time and groups, (3–5) three increasingly restrictive models with additional equality constraints to item/facet parcel intercepts across time and groups (i.e., scalar invariance). For the scalar invariance step, we compared three models to better locate potential measurement invariance violations: equality constraints across (3) time in the change goal group, (4) time in the change goal and comparison group, and (5) both time and groups simultaneously^3^. An illustration of the (self-report facet) model and measurement invariance constraints applied across time and groups is presented in Figure 1. We deemed the first scalar measurement invariance level most relevant for the current study, as it provides an indication if the intervention-related changes generalized to the overarching trait domains or facets. For the test across time in the control group, we did not expect any measurement invariance violations. However, it is possible that some untargeted nuances or facets changed as “side-effects” of the broad underlying intervention-processes, as the comparison group consisted of participants that used similar activities (e.g., self-reflection and behavioral activation), albeit with a different goal. These potential spillover effects might cause invariance violations in the comparison group, as these should not affect the entire trait domain, but only specific components. And finally, we used the last scalar measurement invariance level to examine if we could compare the factor means between the two groups. To estimate the factor means, we used reference group scaling (i.e., constraining the change goal group pre-test factor mean to 0; see Figure 1). We chose this approach as it would allow for a direct interpretation of the factor means as the change compared to the pre-test, or differences of the comparison group to the change goal group at the pre-test.

#### Power analysis

Measurement invariance is generally tested by comparing the model fit between increasingly restrictive models. To ensure that the measurement invariance test was sensitive enough to violations of measurement invariance, we conducted a power analysis for different degrees of scalar measurement invariance violations, sample sizes, and model fit cut-offs. To reflect the structure of our data, we simulated data with three indicators at three measurement occasions and two groups—one “change goal group” with 25–200 participants (in steps of 25) and one “control group” with a fixed number of 300 participants. In the “change goal group,” we modified the population parameters to either reflect no measurement invariance violations or different degrees of non-invariance (i.e., loading or intercept changes in one to four indicators ranging from Δλ = 0.2 to 0.8 or d = 0.1 to 0.5, respectively; see OSF for a detailed description: https://osf.io/q7vgf/). For each combination of sample size and population parameters, we simulated 100 datasets. We then examined the capability of different suggested model fit cutoffs (i.e., ΔCFI < −.002/−.010; ΔRMSEA >.010/.015; ΔSRMR >.010/.015; Chen, 2007; Cheung & Rensvold, 2002; Meade et al., 2008) to either support or reject measurement invariance (i.e., sensitivity and specificity). Detailed results are presented in OSF Tables 1 and 2. Overall, the ΔCFI = −.002 criterion provided the best trade-off between the power to detect violations and robustness against false positives, detecting 73% of simulated scalar measurement invariance violations and only rejecting it in 3% of invariant cases. In the smallest available sample in this study (observer-reported Extraversion with 76/54 participants at the post-test/follow-up), it yielded a power of 87%/70% to detect deviations of d = 0.3 in only one indicator, with power increasing with sample size, number of non-invariant indicators, and degree of non-invariance. For metric invariance, the ΔCFI = −.002 criterion detected 68% of simulated non-invariant cases, but also rejected 17% of invariant models—even in larger samples (e.g., 14% for N = 200 in the change goal group). Using ΔCFI = −.003 slightly improved the balanced accuracy to 64% correctly detected violations and 11% false positives.

**Table 2.** Descriptive statistics of the sample and change goal groups.

	Age	Gender (f)	In a relationship	NE	CO	EX	OP	AG
Full sample	25.35	58.2%	58.7%	2.73	3.50	3.23	3.67	3.85
NE- group	25.30	79.2%	69.7%	3.16	3.68	3.34	3.72	3.80
Control group	25.37	49.0%	53.9%	2.54	3.42	3.18	3.65	3.87
d	−0.01	30.2%	15.8%	1.05	0.38	0.23	0.12	−0.15
p	.922	<.000	<.000	<.000	<.000	.011	.192	.119
CO + group	25.09	47.1%	54.0%	2.51	2.94	3.30	3.84	3.89
Control group	25.26	61.8%	59.9%	2.82	3.67	3.19	3.60	3.84
d	−0.02	−14.7%	−5.9%	−0.49	−1.19	0.17	0.36	0.10
p	.812	.003	.236	<.000	<.000	.069	<.000	.270
EX + group	24.09	39.1%	41.1%	2.60	3.68	2.83	3.47	3.89
Control group	25.59	62.8%	63.0%	2.76	3.45	3.33	3.72	3.84
d	−0.20	−23.7%	−21.9%	−0.26	0.32	−0.77	−0.39	0.11
p	.048	<.000	<.000	.013	.002	<.000	<.000	.335

*Note.* NE-group = decrease in Negative Emotionality change goal group; CO + group = increase in Conscientiousness change goal group; EX + group = increase in Extraversion change goal group; control group = all other participants desiring to change on a different trait domain; d = Cohen’s d/difference in percentage; p = significance value of independent t-test between change goal group and corresponding control group.

We thus used the ΔCFI < −.003/.002 criterion to test for metric/scalar measurement invariance. For the facet models that yielded a higher increase in model misfit than suggested by these cut-offs, we additionally examined the item mean-levels to identify the reason for the measurement invariance violations and consequently the differential effects of the interventions at this level.

## Results

### Selection and attrition

We first examined the differences between the participants in the change goal groups and the corresponding control groups. Descriptive statistics, effect sizes, and independent t-test significance values are presented in Table 2. The largest differences between the groups could be found for the target trait domains, with participants reporting lower levels of the trait domain if they wanted to increase (i.e., d = −1.19/−0.77 for Conscientiousness/Extraversion in the corresponding change goal group), and higher levels if they wanted to decrease on a trait (i.e., d = 1.05 for Negative Emotionality in the corresponding change goal group). We also found some small differences between the groups with respect to the traits not chosen as the change goal. However, these generally showed that participants had slightly higher (or lower in the case of Negative Emotionality) levels on the other trait domains, which can be explained by the comparison group including those participants who wanted to change on the other traits—and thus had lower (or higher in the case of Negative Emotionality) levels to begin with. With respect to age, the largest differences were found for the increase in Extraversion group, which was generally younger (d = −0.20) than the remaining sample. With respect to differences in gender and relationship, the Negative Emotionality decrease group was predominantly female (79.2% compared to 49.0% in the remaining sample) and in a relationship (69.7% compared to 53.9%). In contrast, the Extraversion increase group consisted mostly of males (62.8% compared to 39.1%) and singles (58.9% compared to 37.0%).

Of the 1523 participants who filled out the pre-test, only 36.2% also filled out the post-test. These participants were higher on self- and other-reported Conscientiousness (self-report: d = 0.15; other-report d = 0.29; *p* < .001) than participants who dropped out of the study but did not differ on any other trait-domain. Moreover, a larger proportion of the sample that provided responses on at least two occasions was female (58.1% compared to 48.4%; *p* < .001) and in a relationship (58.7% compared to 50.1%; *p* = .002). We did not find any differences in age between the two groups.

### Model fit, measurement invariance, and reliability

#### Self-report

Reliability and model fit across measurement invariance levels for the self-report models are presented in Table 3. All models achieved acceptable absolute model fit across all measurement invariance levels. Reliability (McDonald’s omega) was on average ω = .77 for all self-report facets and adequate (ω ≥ .70) for all facets and trait domains except for the responsibility facet (ω = .63).

**Table 3.** Self-report data model fit, measurement invariance, and reliability.

Negative Emotionality change goal																				
	Anxiety ω = .70					Depression ω = .80					Emotional Volatility ω = .79					Negative Emotionality (domain) ω = .81				
MI level	df	χ^2^	CFI	RMSEA	SRMR	df	χ2	CFI	RMSEA	SRMR	df	χ2	CFI	RMSEA	SRMR	df	χ2	CFI	RMSEA	SRMR
configural	78	136	.970	.052	.040	78	199	.959	.075	.060	78	111	.987	.039	.035	30	40	.996	.035	.030
Metric	93	155	.968	.049	.047	93	228	.954	.072	.069	93	122	.988	.034	.043	40	48	.997	.027	.036
Sca. (change)	**99**	**170**	**.964**	**.051**	**.048**	**99**	**271**	**.941**	**.079**	**.069**	99	131	.987	.034	.043	**44**	**62**	**.994**	**.039**	**.034**
Sca. (contr.)	**105**	**188**	**.958**	**.053**	**.050**	**105**	**343**	**.919**	**.091**	**.073**	105	135	.988	.032	.044	48	69	.993	.040	.034
Sca. (groups)	**108**	**197**	**.955**	**.055**	**.055**	108	344	.919	.089	.074	108	146	.985	.036	.046	**50**	**100**	**.982**	**.060**	**.051**

Conscientiousness change goal																				
	Organization ω = .88					Productiveness ω = .79					Responsibility ω = .63					Conscientiousness (domain) ω = .79				
MI level	df	χ2	CFI	RMSEA	SRMR	df	χ2	CFI	RMSEA	SRMR	df	χ2	CFI	RMSEA	SRMR	df	χ2	CFI	RMSEA	SRMR
configural	78	129	.987	.049	.035	78	163	.967	.064	.054	78	96	.989	.029	.037	30	25	1.000	.000	.020
Metric	93	152	.985	.049	.047	93	183	.965	.060	.063	93	114	.987	.029	.048	40	40	1.000	.000	.028
Sca. (change)	99	159	.985	.048	.049	99	188	.966	.058	.064	**99**	**142**	**.974**	**.040**	**.050**	**44**	**80**	**.987**	**.055**	**.039**
Sca. (contr.)	105	169	.984	.048	.050	**105**	**204**	**.962**	**.060**	**.065**	**105**	**171**	**.959**	**.049**	**.056**	**48**	**138**	**.968**	**.084**	**.046**
Sca. (groups)	**108**	**186**	**.981**	**.052**	**.059**	108	211	.960	.060	.065	**108**	**189**	**.950**	**.053**	**.064**	50	140	.968	.082	.051

Extraversion change goal																				
	Sociability ω = .80					Assertiveness ω = .82					Energy level ω = .73					Extraversion (domain) ω = .77				
MI level	df	χ2	CFI	RMSEA	SRMR	df	χ2	CFI	RMSEA	SRMR	Df	χ2	CFI	RMSEA	SRMR	df	χ2	CFI	RMSEA	SRMR
Configural	78	146	.980	.056	.038	78	136	.983	.052	.038	78	164	.967	.063	.058	30	36	.998	.027	.022
Metric	93	162	.980	.052	.050	93	144	.985	.045	.043	93	185	.964	.060	.062	40	58	.995	.040	.056
Sca. (change)	99	175	.978	.053	.054	**99**	**163**	**.981**	**.049**	**.044**	99	196	.963	.060	.064	**44**	**123**	**.977**	**.081**	**.045**
Sca. (Contr.)	**105**	**212**	**.969**	**.061**	**.059**	**105**	**185**	**.976**	**.053**	**.047**	**105**	**223**	**.954**	**.064**	**.067**	48	129	.976	.078	.046
Sca. (Groups)	108	213	.969	.060	.057	108	186	.977	.051	.047	**108**	**249**	**.945**	**.069**	**.067**	50	133	.976	.078	.047

*Note.* ω = McDonald’s factor saturation omega; df = degrees of freedom; χ2 = chi-square value; CFI = Comparative Fit Index; RMSEA = Root Mean Square Error of Approximation; SRMR = Standardized Root Mean Square Residual; MI-level = measurement invariance level; configural = no additional constraints across time or groups; metric = factor loading equality constraints across time and groups; sca. (change) = additional item/facet parcel intercept equality constraints across time in the change goal group; sca. (contr.) = additional item/facet parcel intercept equality constraints across time in the control group; sca. (groups) = additional item/facet parcel intercept equality constraints across the two groups. Values marked in bold indicate ΔCFI < −.002 for scalar measurement invariance.

For the metric measurement invariance level, increases in model misfit did not exceed ΔCFI < −.003. With respect to scalar invariance across time in the change goal group, four out of nine facets resulted in notable increases in model misfit, with smaller increases for anxiety (N; ΔCFI = −.004) and assertiveness (E; ΔCFI = −.004), but more severe violations for depression (N; ΔCFI = −.013) and responsibility (C; ΔCFI = −.013). All three trait domains showed differential patterns of change in the corresponding change goal group, most strongly for Extraversion (ΔCFI = −.018) and Conscientiousness (ΔCFI = −.013), and only to a smaller degree for Negative Emotionality (ΔCFI = −.003). Surprisingly, measurement invariance violations were more common in the combined comparison groups (seven non-invariant facets across time and the Conscientiousness trait domain). Because these groups consisted of all participants working on other trait domains, this suggests that some components of un-targeted traits changed as “side-effects” of the intervention.

#### Observer-report

Reliability and model fit across measurement levels for the observer-report models are presented in Table 4. All models achieved acceptable absolute model fit across all measurement invariance levels. Reliabilities were adequate for Negative Emotionality (ω = .82) and Conscientiousness (ω = .77), but lower than acceptable levels for Extraversion (ω = .63). For metric measurement invariance, we found some increases in model misfit for the Conscientiousness (ΔCFI = −.009) and Extraversion (ΔCFI = −.008) trait domains. For the Extraversion trait domain, we also found issues with scalar invariance across time in the change goal group (ΔCFI = −.007).

**Table 4.** Observer-report data model fit, measurement invariance, and reliability.

Negative Emotionality change goal															
	Negative Emotionality ω = .82					Conscientiousness ω = .77					Extraversion ω = .63				
MI level	df	χ^2^	CFI	RMSEA	SRMR	df	χ2	CFI	RMSEA	SRMR	df	χ2	CFI	RMSEA	SRMR
Configural	30	28	1.000	.000	.035	30	52	.985	.067	.038	30	40	.993	.043	.030
Metric	40	40	1.000	.000	.043	40	76	.976	.073	.068	40	60	.985	.055	.057
Sca. (change)	48	44	1.000	.000	.042	48	85	.975	.069	.069	**48**	**79**	**.978**	**.061**	**.059**
Sca. (contr.)	44	42	1.000	.000	.042	44	84	.974	.074	.069	44	73	.979	.062	.058
Sca. (groups)	50	45	1.000	.000	.045	**50**	**96**	**.969**	**.075**	**.066**	50	81	.978	.060	.062

*Note.* ω = McDonald’s factor saturation omega; df = degrees of freedom; χ2 = chi-square value; CFI = Comparative fit index; RMSEA = root mean square error of approximation; SRMR = standardized root mean square residual; MI-level = measurement invariance level; configural = no additional constraints across time or groups; metric = factor loading equality constraints across time and groups; sca. (change) = additional item/facet parcel intercept equality constraints across time in the change goal group; sca. (contr.) = additional item/facet parcel intercept equality constraints across time in the control group; sca. (groups) = additional item/facet parcel intercept equality constraints across the two groups. Values marked in bold indicate ΔCFI < −.002 for scalar measurement invariance.

### Negative emotionality facet and item means across time and groups

**Figure 2.**
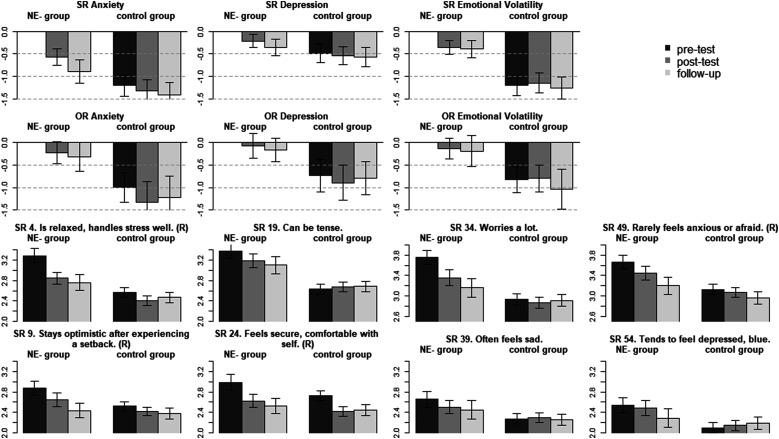
Negative emotionality facet and item mean-levels across time and groups. *Note*. SR = self-report; OR = observer-report; NE-group = decrease in Negative Emotionality change goal group; control group = all other participants desiring to change on a different trait domain; SR 4 to 54 = item number from the self-report BFI-2. (R) = item was reverse coded. Bars represent standardized differences to the change goal group at the pre-test as reference (i.e., reference factor mean set to 0). Error bars represent the 95% confidence interval. Item labels are taken from Soto and John (2017a).

The Negative Emotionality change goal group was the largest in this study, with 168 participants desiring a decrease on this trait. The Negative Emotionality facet means, as well as item means of the anxiety and depression facet, are presented in Figure 2. At the beginning of the intervention, participants from the Negative Emotionality change group (self-) reported higher anxiety (*d* = 1.20; *p* < .001), depression (*d* = 0.49; *p* < .001), and emotional volatility (*d* = 1.20; *p* < .001) than participants from the other groups. In the observer-report data, participants from the Negative Emotionality change group were rated to have higher anxiety (*d* = 0.99; *p* < .001), depression (*d* = 0.74; *p* < .001) and emotional volatility (*d* = 0.82; *p* < .001) than other participants in the study. Over the course of the intervention, all three self-reported facet levels decreased in the change goal group (post-test/follow-up: anxiety: *d* = −0.57/−0.89; *p* < .001; depression: *d* = −0.22/−0.36; *p* = .004/*p* < .001; emotional volatility: *d* = −0.36/−0.39; *p* < .001). The lack of measurement invariance across time for Negative Emotionality could thus be explained by the predominant decrease in anxiety. Despite some descriptively substantial effects (e.g., anxiety: *d* = −0.22/−0.31), none of the observer-reported changes were significant, arguably due to the lower power in the smaller samples.

The self-report anxiety and depression facet model suffered from a lack of measurement invariance. As indicated by the relatively small increase in model misfit for anxiety (ΔCFI = −.004), the differences in item-level change were relatively small, with responses to “Can be tense” (item 19) and “Rarely feels anxious or afraid” (item 49; reverse coded) changing to a smaller degree than responses to the other two items (average unstandardized difference between post- and pre-test: −0.28 vs. −0.42). For depression (ΔCFI = −.013), changes were strongest for the items “Stays optimistic after experiencing a setback” and “Feels secure, comfortable with self” (item 9/24; reverse coded; unstandardized difference between post- and pre-test: −0.23/−0.36; *p* = .020/.004), whereas the other two items did not change significantly during the intervention.

### Conscientiousness facet and item means across time and groups

**Figure 3.**
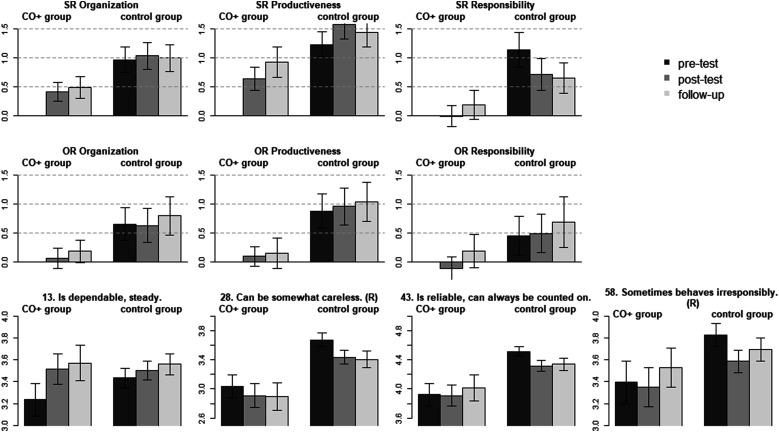
Conscientiousness facet and item mean-levels across time and groups. *Note*. SR = self-report; OR = observer-report; CO + group = increase in Conscientiousness change goal group; control group = all other participants desiring to change on a different trait domain; SR 13 to 58 = item number from the self-report BFI-2. (R) = item was reverse coded. Bars in the first two rows (facets and trait domain) represent standardized differences to the change goal group at the pre-test as reference (i.e., reference factor mean set to 0). For the bottom row (nuances) unstandardized item means are reported. Error bars represent the 95% confidence interval. Item labels are taken from Soto and John (2017a).

A total of 140 participants wanted to increase in Conscientiousness. The facet and responsibility item means are presented in Figure 3. At the beginning of the intervention, participants from the change group (self-)reported substantially smaller organization (*d* = −.97; *p* < .001), productiveness (*d* = −1.23; *p* < .001), responsibility (*d* = −1.14; *p* < .001) than the other groups. In the observer-report data, participants from the change group were reported to have lower organization (*d* = −0.66; *p* < .001), productiveness (*d* = −0.88; *p* < .001), and responsibility (*d* = −0.45; *p* = < .001) levels than the other participants in the study. Over the course of the intervention, self-reported increases were found for organization (post-test/follow-up: *d* = 0.42/0.49; *p* < .001) and productiveness (post-test/follow-up: *d* = 0.64/0.93; *p* < .001), but not responsibility (post-test/follow-up: d = −0.09/0.19; *p* = .920/.148). The intervention thus seemed to affect the first two facets most strongly, whereas responsibility did not change significantly due to the intervention—explaining the strong violation of measurement invariance across time for self-reported Conscientiousness (ΔCFI = −.013). Observers did not report any significant changes in the facet mean-levels during the intervention

The self-report responsibility facet model suffered from a lack of measurement invariance (ΔCFI = −.013). The item means (see Figure 3) show that only being steady and dependable increased during the intervention (item 13; unstandardized post-test/follow-up to pre-test difference = 0.28/0.33; *p* = .007/.003), whereas being careless (item 28), reliable (item 43), or irresponsible (item 58) did not significantly differ between the beginning of the intervention and later measurement occasions.

### Extraversion facet and item means across time and groups

**Figure 4.**
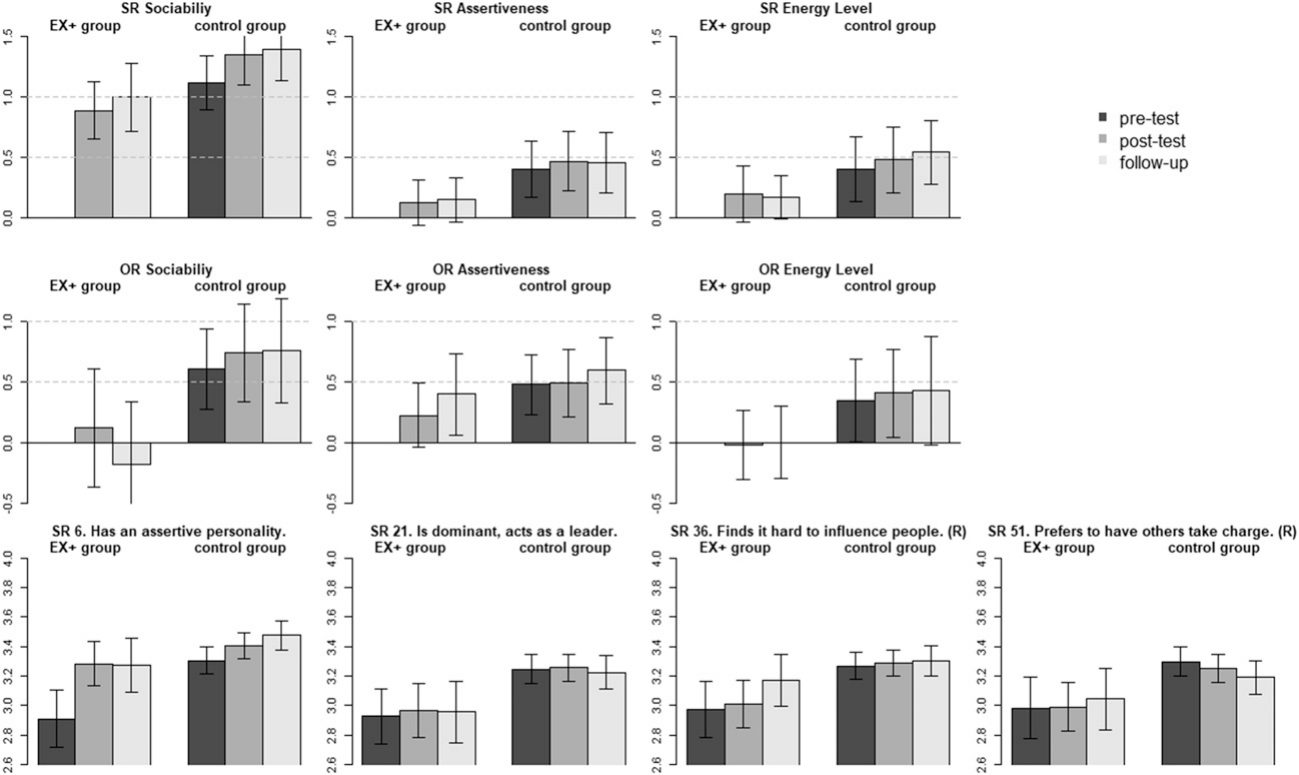
Extraversion facet and item mean-levels across time and groups. *Note*. SR = self-report; OR = observer-report; EX + group = increase in Extraversion change goal group; control group = all other participants desiring to change on a different trait domain; SR 6 to 51 = item number from the self-report BFI-2. (R) = item was reverse coded. Bars represent standardized differences to the change goal group at the pre-test as reference (i.e., reference factor mean set to 0). Error bars represent the 95% confidence interval. Item labels are taken from Soto and John (2017a).

The Extraversion change goal group was the third largest, with 110 participants wanting to increase on this trait. The corresponding facet means are presented in Figure 4. At the beginning of the intervention, participants from the Extraversion change group (self-)reported substantially lower levels of sociability (*d* = −1.11; *p* < .001), assertiveness (*d* = −0.40; *p* = .001), and energy-level (*d* = −0.40; *p* = .003) than the comparison group. In the observer-report data, participants from the change group were also reported to have lower levels on sociability (*d* = −0.60; *p* < .001), assertiveness (*d* = −0.48; *p* < .001), and energy level (*d* = −0.35; *p* = .047) than the other participants in this study.

Self-reported increases on the facet mean levels between the pre- and post-test or follow-up were very large for sociability (*d* = 0.89/1.00; *p* < .001), but non-significant for assertiveness (*d* = 0.12/0.15; *p* = .189/.113) and energy-level (*d* = 0.20/0.17; *p* = .097/.061). As such, the intervention seemed to have the strongest effect on participants self-perceived sociability—explaining the severe violation of scalar measurement invariance for Extraversion across time (ΔCFI = −.018). Whereas self-reported assertiveness did not increase significantly, the observers reported an increase in participants’ assertiveness between the pre-test and follow-up (*d* = 0.40; *p* = .019), but no significant change in the other facets.

For the assertiveness facets, we found a violation of scalar measurement invariance across time (ΔCFI = −.004). The item means (see Figure 4) show that participants in the change goal group increased their agreement with “Has an assertive personality” (item 6; unstandardized post-test/follow-up to pre-test difference = 0.37/0.36; *p* = .003/.007), but remained stable with respect to the other items.

## Discussion

Personality change interventions and the use of digital applications for intervention efforts have recently attracted attention in the field of personality development (Allemand & Flückiger, 2022). Recent intervention work provided initial evidence for the effectiveness of a three-month coaching intervention with a digital coach (Stieger et al., 2021). The present study used the same dataset to explore the effects of the intervention on personality facets and nuances with the goal to provide a more fine-grained picture of the intervention effects using both self- and observer-reports. Personality intervention studies typically focus on mean-level differences at the trait domain level, but do not account for potential differences in change at the facet or nuance level. The main objective of this study was to examine whether a three-month digital-coaching intervention with the goal of changing the trait domain levels (Stieger et al., 2021) affected all lower-level constructs in a similar fashion. We demonstrated how measurement invariance testing can be used to identify a heterogeneity in the mean-level change in the broad and hierarchical trait domains and examined facet-level changes during the intervention.

### Measurement invariance and mean-level change

Measurement invariance testing is well established in the context of personality development studies (e.g., Allemand et al., 2007; Nye et al., 2016; Olaru et al., 2018). Although it is generally seen as a prerequisite for the examination of changes or differences in the factor means, violations thereof can also provide useful information on differential change in the traits. This can be particularly interesting to judge how the traits are affected by the intervention (Könen & Karbach, 2021; Putnick & Bornstein, 2016). In this study, we were primarily interested in mean-level change and thus focused on scalar measurement invariance, but in cases in which the intervention is assumed to change the structure of a construct metric measurement invariance can also be focused on. A violation of metric measurement invariance (i.e., equal factor loadings) would suggest that the relationship between the latent and manifest variables has changed during the intervention. For example, an intervention targeting a single behavior or facet specifically might lead to a disruption of the association with other indicators of the trait domain, which might be detected through violations of metric measurement invariance. In the current study, we found no effects on the covariance structure for the self-reports, but some differences in the observer-report factor loadings (i.e., Extraversion and Conscientiousness loadings). However, because these deviations were only found for the observer-reports with generally smaller sample sizes and partly changing raters, we think it is unlikely that the factor loading differences reflect actual changes to the structure of the trait domains.

For all three self-report trait domains, we found differences in the mean-level change at the facet level (i.e., violations of scalar measurement invariance). Most notably, self-reported sociability showed a very strong increase over the course of the intervention, whereas increases in the other Extraversion facets of assertiveness and energy-level were small and non-significant. For Conscientiousness, participants in the change goal group reported moderate increases in organization and productiveness, but no increase in responsibility. For Negative Emotionality, all participants reported decreases in all three facets over the course of the intervention, but most strongly in anxiety. In the observer-reports, we only found a lack of scalar measurement invariance for Extraversion, with observers reporting an increase in assertiveness but no other facet. Overall, observers did not report any significant changes in the Negative Emotionality or Conscientiousness facets, which can explain why these trait domains were scalar measurement invariant across time.

For four out of the nine self-reported facets, the items also showed differential rates of change during the intervention. For anxiety, participants reported improvements on all items but most strongly in their ability to handle stress and worry less. Regarding the depression facet, participants reported to become more optimistic after setbacks and more comfortable with themselves, but not to decrease in the frequency of feeling sad or depressed. This suggests an improvement in self-acceptance and coping, instead of a direct reduction of the frequency of sadness. Being more comfortable with oneself also improved in the comparison group (see Figure 2), suggesting that the intervention improved self-acceptance independently of the chosen change goal. For assertiveness and responsibility, item-level responses generally did not change with the exception of one item per facet. Interestingly, participants scored higher on “has an assertive personality” after the intervention, but did not increase on any of the other assertiveness items. This might suggest that participants interpreted assertiveness differently than is operationalized by the BFI-2 items of that facet, or changed in some aspects of assertiveness not measured by the scale. And finally, participants from the Conscientiousness change goal group reported to become more steady and dependable, but did not rate themselves higher on any other responsibility item.

Taken together, these findings suggest considerable heterogeneity in the change processes during the intervention. Because most personality intervention or development studies focus on changes in trait domain scale scores, such effects are potentially overlooked and change in only one facet, or some items may be interpreted as a (weaker) change in the entire trait domain. Apart from an impact on the effect size, such overlooked invariance violations can also affect the interpretation of the found effects. For instance, we found that the “depression” facet mean showed a decrease during the intervention—without any mean-level changes in the sadness or depressive mood item. As such, heterogeneous scales and jingle-jangle fallacies in the scale labels can further exacerbate issues of overlooked measurement invariance violations.

### Potential reasons for differences in change

Differences in the rate of change were particularly strong for the facets of Extraversion and Conscientiousness, which might have been caused by differences in the motivation to change on the sub-traits. Personality change goals have been shown to be positively associated with the degree of self-regulated change (Hudson et al., 2020; see also Hennecke et al., 2014). One study comparing the desire to change in the facets of the BFI-2 showed that—when asked to pick only three change goals—participants preferred sociability over assertiveness and energy-level, productivity over organization and responsibility, and (lower) anxiety and (lower) depression over (lower) emotional volatility (Sun & Goodwin, 2020). In line with this, we found the strongest changes for anxiety, sociability, productivity, and organization. Participants in personality intervention studies seem to primarily choose traits that are most strongly related to relevant life outcomes (e.g., higher life satisfaction, better work performance, and better social connectedness; see also Hudson & Fraley, 2017; Hudson & Roberts, 2014). Within the trait domain interventions, they might thus make a higher effort to change the facets they deem most desirable or most relevant to the achievement of their goals and focus less on other sub-traits. For example, the Extraversion change goal group of this study consisted mostly of male singles who were particularly low on sociability and less satisfied with their sexual life (see Stieger, Eck, et al., 2020). Their primary goal for participating in this group might thus have been to increase their sociability levels to increase their chances to find a (sexual) partner, but not necessarily to increase their assertiveness or energy-level.

Asking participants what traits they want to change in more detail, as well as the reasons for their change goals, might help understand potential differences in the mean-level change between facets. A change goal inventory with the same level of detail as the scale used to evaluate the intervention (e.g., a change goal BFI-2) could be used to examine whether the facet mean-level changes are proportional to the desire to change on these facets. As most participants generally report a desire to change on several traits (e.g., Hudson & Roberts, 2014; Sun & Goodwin, 2020; Thielmann & de Vries, 2021), but can effectively only work on one or a few traits at a time, a forced-choice or ranking measure (see e.g., Sun & Goodwin, 2020) could be used to determine which traits participants are most likely to prioritize during the intervention. Asking participants about what exactly they wish to achieve by changing their personality traits (e.g., higher well-being, better job performance, and higher likelihood of finding a partner) and how important those goals are to them can further help understand differences in the intervention effects.

The current intervention (see also Stieger et al., 2018, 2021) used a broad range of intervention activities (e.g., self-reflection, behavioral activation, implementation intentions, and resource activation). This was a strength of the current intervention as it would target the trait domains more comprehensively than smaller sets of activities. To examine which activities are particularly effective at changing personality traits, intervention studies could compare the effects of subsets of the activities, such as implementation intentions or behavioral activation tasks (Hudson et al., 2019; Hudson & Fraley, 2015) to self-reflection and resource activation. By comparing the effects across different trait domains or specifically targeted facets (e.g., openness to action vs. self-discipline; Stieger, Wepfer, et al., 2020), it can be better understood whether specific sets of activities are better suited to change personality traits in general, or some traits in particular.

However, when doing so, it is important to also consider the way the traits are operationalized or measured. Although the Big Five trait domains (and consequently smaller sub-traits) are conceptually understood to explain or represent stable patterns of affect, behaviors, and cognition, these content domains are confounded with traits in personality measures (Pytlik Zillig et al., 2002; Wilt & Revelle, 2015). For instance, Wilt and Revelle (2015) found that Neuroticism (or Emotional Stability) is primarily measured with affect items, Conscientiousness and Extraversion are predominantly assessed with behavioral items, and Openness with items from the cognitive content domain. Such differences in item content may explain differences in the rate of change or effects of various intervention activities (e.g., behavioral activation primarily affecting behavioral items). Although these can also be detected with measurement invariance tests, the capability to do so is limited by unbalanced scales (e.g., if the majority of items in one trait domain or facet measure behaviors). Broader and more balanced scales are thus needed to better understand personality change, both in the context of intervention studies and personality development in general.

### Limitations and future directions

With respect to the sample and design, there are a several limitations of the present work that deserve attention in future research. First, we used participants from the other change goal groups as active control groups in the present study. As such, the intervention and comparison sample were not randomly allocated and showed strong mean-level differences at the beginning of the study. We did not use the available waitlist passive control group (see Stieger et al., 2021) because of the low sample size and shorter time span covered (i.e., 4 weeks). Because the models used assume similar time spans between measurements, the passive control group would not have been directly comparable. One limitation due to the lack of a passive control group was the ability to control for regression to the mean for the change goal groups. However, based on the same dataset, Stieger and colleagues (2021) examined mean-level changes in the randomly allocated passive control group. Contrary to a regression to the mean effect, they reported no trait domain changes in the direction of the change goals in any of the passive control groups (i.e., separated by change goals). As such, we also assume that the facet level changes were similarly unaffected by this artifact.

Second, we asked participants to indicate their change goal by choosing one of nine descriptions. This approach was chosen to identify the strongest change desire for each participant. However, we did not know how strong the desire to change was overall, and whether there were differences in the desire to change between facets of the chosen trait domain. The degree of trait change is generally reported to be proportional to the participants’ desire to change on the trait (e.g., Hudson et al., 2020). Using a change goal inventory based on the BFI-2 (see e.g., Sun & Goodwin, 2020) would have allowed us to further examine this association at the facet and item level, as well as potentially explain the heterogeneity in change based on differences in the motivation to change facets of a common trait.

Third, while we also used observer-reports to examine the intervention effects, the reported effects were small and insignificant in most cases. Observers in this study were generally very close to the targets and potentially knew them for several years (or even the entire life in the case of parents). As such, observations over the period of three or six months might not have been enough to update their pre-existing view of the targets. Changes in the behavior of the target might have been interpreted as temporary fluctuations from the normal behaviors instead of the more substantial change that participants reported themselves. Another potential explanation for the lack of observer-reported mean-level changes is that targets might not have changed their behavior as much during the interactions with the observers, as the targeted traits were potentially irrelevant for the relationship (e.g., productivity and organization) or used primarily in interactions with new acquaintances (e.g., sociability). To judge the efficacy of personality interventions, it is essential to include more measures beyond self-reports. Observer-reports are a relevant source of information for the evaluation of the effects of personality interventions but may need longer observation periods and groups of observers with a high frequency of interactions and relatively short duration of acquaintance prior to the intervention. For example, college students could be rated by their new peers or friends at university, and non-college samples by their work colleagues. Another way to improve the usefulness of observer-reports in the context of personality intervention studies could be to select observers based on the intervention group, for example, work colleagues for Conscientiousness interventions, or romantic partners for Negative Emotionality or Agreeableness interventions. By ensuring that the traits to be changed are particularly relevant for the interactions of the target and partner, potential changes might be easier to detect through observer-reports.

Fourth, only around 36% of the initial sample also providing responding to the post-test. All intervention activities and assessments were administered digitally, without any direct contact between the participants and researchers. This is a strength of the intervention, as it can be easily applied to larger groups of people, and shows that participants achieved personality change without a direct interaction between participants and a human coach. To decrease the rate of attrition in future digital-coaching personality intervention studies, some form of contact between researchers and participants could be established (e.g., administering some activities in the lab).

Fifth, we used the BFI-2 for the self-reports, and the shorter version BFI-2-S for the observer reports. As such, the comparability between the two assessment forms was reduced. The shorter observer-report inventory might have also reduced the potential to identify observed personality changes, as both construct coverage and measurement precision are negatively affected by lower item numbers.

And finally, the choice of measurement instrument can affect the capability to detect possible changes and measurement invariance violations in the context of personality change studies (e.g., Olaru & Jankowsky, 2022). We chose the BFI-2, because it provides a broad measure of the personality trait domains with the three most central facets per trait domain. Compared to shorter inventories, potentially without a facet structure, the BFI-2 should be better able to capture changes in the personality traits, and the heterogeneity thereof. However, future studies could use much broader inventories with more items and facets (e.g., NEO-PI-R; Costa & McCrae, 2008) to examine these changes in more details.

### Conclusion

The current study provided new knowledge about the changeability of personality facets and nuances in response to participation in a digital-coaching intervention. We demonstrated the relevance of measurement invariance testing and the analysis of more fine-grained trait levels than the trait domains in the context of personality intervention studies. Using measurement invariance testing, we detected considerable heterogeneity in the self-reported mean-level change for Negative Emotionality, Extraversion, and Conscientiousness. Most notably, self-reported changes were strongest for anxiety (Negative Emotionality), sociability (Extraversion), productiveness, and organization (Conscientiousness). Furthermore, changes in four of the nine facets in the self-reports represented changes in only some item means. For the observer reports, we only found scalar measurement invariance violations for Extraversion. Taken together, this study provides further and more fine-grained evidence for the efficacy of a digital-coaching personality change intervention (Stieger et al., 2021), but also shows the need for more precise analytical and measurement approaches that account for the complexity of personality traits and interventions.

## Supplemental Material

Supplemental Material - Personality change through a digital-coaching intervention: Using measurement invariance testing to distinguish between trait domain, facet and nuance change Supplemental Material for Personality change through a digital-coaching intervention: Using measurement invariance testing to distinguish between trait domain, facet and nuance change by Gabriel Olaru, Mirjam Stieger, Dominik Rüegger, Tobias Kowatsch, Christoph Flückiger, Brent W Roberts, and Mathias Allemand in European Journal of Personality

Supplemental Material - Personality change through a digital-coaching intervention: Using measurement invariance testing to distinguish between trait domain, facet and nuance change Supplemental Material for Personality change through a digital-coaching intervention: Using measurement invariance testing to distinguish between trait domain, facet and nuance change by Gabriel Olaru, Mirjam Stieger, Dominik Rüegger, Tobias Kowatsch, Christoph Flückiger, Brent W Roberts, and Mathias Allemand in European Journal of Personality

## Notes

1. The design of the digital intervention study included a randomized control trial with an intervention condition and a waitlist control condition with a 4-week waiting period followed by the same intervention as in the intervention condition (see Figure 1 in Stieger et al., 2021). Moreover, the intervention condition was oversampled and included 2/3 of all participants. Due to the smaller size of the personality change goal groups in the waitlist control condition and the time-limited waiting period, we pooled both conditions for this paper and focused on the three largest change goal groups. For each of the change goal groups, we used participants that desired to change on a different Big Five factor as the comparison groups.

2. A total of 134 (24.3%) participants were part of the smaller change goal groups.

3. Because the observer-report facets only had two items, and would thus only add one constraint per model, we directly constrained all item intercepts across time and groups in one step.
